# Whole-Genome Analysis and Lignin Degradation Characterization of Termite-Derived *Bacillus cereus* BC-8

**DOI:** 10.3390/microorganisms14010054

**Published:** 2025-12-26

**Authors:** Xingbo Zhang, Jingtao Li, Yue Hu, Zhanbo Cai, Nan Li, Runsen Xue, Zexuan Mo, Chenghao Yang, Yuhui Yang

**Affiliations:** 1 College of Life and Health Sciences, Hainan University, Haikou 570228, China; 15105690350@163.com; 2 College of Tropical Agriculture and Forestry, Hainan University, Haikou 570228, China; 15839127638@163.com (J.L.); 15289852251@163.com (Y.H.); 17762524946@163.com (Z.C.); 13361319390@163.com (N.L.); 18435241766@163.com (R.X.); mozexuan1@163.com (Z.M.); mamoru1927@outlook.com (C.Y.)

**Keywords:** *Bacillus cereus* BC-8, lignin degradation, structural analysis, whole-genome sequencing, functional gene annotation

## Abstract

Lignin is one of the primary biomass resources in nature; however, its highly stable structure makes it difficult to degrade and utilise. As efficient decomposers of lignocellulosic biomass, termites rely on their gut microbiota for digestion. Consequently, termite guts harbour abundant and specialized lignin-degrading microorganisms. In this study, we isolated a bacterium from the termite gut and identified it as *Bacillus cereus* BC-8. The laccase activity of *B. cereus* BC-8 reached the maximum of 87.8 U/L at 72 h, and the lignin degradation rate reached 33.66% within 7 days. Furthermore, we analyzed the structural changes in lignin after treatment with this bacterial strain. Field emission scanning electron microscopy observations revealed that the surface structural integrity of lignin was significantly disrupted after treatment. Fourier transform infrared spectroscopy analysis indicated that *B. cereus* BC-8 affected the side chains and aromatic skeleton structures of lignin. Thermogravimetric analysis further revealed that *B. cereus* BC-8 disrupted the primary inter-unit β-O-4 ether bonds of lignin. Whole-genome sequencing of *B. cereus* BC-8 revealed a genome length of 5,374,773 bp and a GC content of 35.34%. Functional gene annotation revealed that the *B. cereus* BC-8 genome contains genes encoding various lignin-degrading enzymes (laccase, cytochrome P450, and vanillin oxidase) and their auxiliary factors, along with the phenylalanine and benzoic acid metabolic pathways, which are associated with lignin degradation. In conclusion, *B. cereus* BC-8 can break down the side chains, aromatic skeletons, and β-O-4 ether bonds of lignin molecules, demonstrating excellent lignin degradation ability. At the molecular level, this study elucidates the key genes and metabolic pathways related to lignin degradation in the genome of *B. cereus* BC-8.

## 1. Introduction

Lignin is a general term for aromatic polymers produced by the oxidative coupling of 4-hydroxybenzophenones, a major component of lignocellulose [1]. As the most abundant renewable phenolic polymer on Earth [2], lignin constitutes the primary component of plant secondary cell walls and plays a crucial role in maintaining the structural integrity of cellulose, hemicellulose, and pectin matrices [3]. At the cellular and molecular levels, lignin serves as a core factor in biomass stress resistance by regulating permeability and enhancing cellular mechanical strength, enabling resistance to external microbial and chemical erosion [4]. However, the structural stability of lignin poses challenges for its efficient utilisation. Research on lignin degradation has increased in recent years, and microbial degradation is a promising avenue for the valorisation of lignin. Microbial enzymes can specifically target and bind lignin, degrading it into aromatic monomers and oligomers [5]. Among these enzymes, laccases [6] and dye decolorising peroxidases [7] are widely present in lignin-degrading bacteria. Known lignin-degrading bacteria are primarily distributed within the *Actinobacteria* [8], *Basidiomycota* [9], and *Proteobacteria phyla* [10], with the genera *Rhodococcus* [11], *Streptomyces* [12], *Pseudomonas* [13], and *Bacillus* [14] being the most common. Notably, increasingly diverse sources of lignin-degrading bacteria are emerging. Nadia et al. [15] screened decaying wood samples and identified multiple bacterial strains with lignin-degrading capabilities, including *Serratia*, *Enterobacter*, *Raoultia*, and *Bacillus*. All these strains exhibited laccase activity and could survive in a medium with lignin as the sole carbon source. Zhu et al. [16] isolated an alkaliphilic bacterium from South China Sea sediments that can degrade lignin via the gentianic acid, benzoic acid, and β-ketohexanedioic acid pathways. Kumar et al. [17] isolated a strain of *Bacillus cereus* from activated sludge. The degradation rate of kraft lignin reached 89%, and the decolorization rate of aniline blue reached 40%. Yang et al. [18] isolated 19 potential strains from rotten wood samples in Qinling Mountain. Using aniline blue decolorization medium for screening, they obtained one strain with robust lignin-degrading enzyme-producing activity, which was identified as *Burkholderia* sp. This strain achieved a lignin degradation rate of 16.74% within 15 days. The above studies indicate that lignin-degrading bacteria are abundant and widely distributed. Among the lignin-degrading bacteria, *Bacillus* has demonstrated the most prominent effect on lignin degradation, with 89% lignin degradation by *Bacillus cereus* [19]. Therefore, it has important value for the development of *Bacillus cereus* strain resources.

Termites are model organisms for the biological pretreatment of lignocellulose as their intestines harbour rich bacterial communities [20,21]. Related studies have shown that termite intestines contain 10^6^–10^8^ bacteria [22], including a large number of lignin-degrading bacteria. Therefore, termite intestines are considered a natural reservoir for lignocellulose-degrading bacteria [23]. Edimar et al. [24] analyzed macrogenomic and transcriptomic data and found that the gut microbiota of termites (containing bacteria from the *Bacillus*, *Enterobacter*, and *Salmonella* genera) are rich in genes encoding lignin-degrading enzymes, such as laccase, peroxidase, glutathione oxidase, and cytochrome P450. In recent years, numerous researchers have screened lignin-degrading bacteria from termites. Tsegaye et al. [25] isolated two lignin-degrading bacteria from *Cryptotermes*, namely *Bacillus* BMP01 and *Ochrobactrum* sp. BMP03, both of which exhibit degradation activity towards lignin and cellulose. However, existing studies on lignin-degrading bacteria derived from termites have primarily focused on characterizing their degradation properties, and the underlying molecular mechanisms involved remain to be further explored [26].

In this study, a bacterium with lignin-degrading ability was isolated from the gut of termites and identified as *B. cereus* BC-8. The lignin degradation activity of this bacterium was systematically elucidated by analyzing the structural and property changes in lignin after *B. cereus* BC-8 treatment. Furthermore, whole-genome sequencing and functional gene annotation results revealed the biological characteristics of *B. cereus* BC-8 at the molecular level and the regulatory mechanisms underlying its lignin degradation capabilities, providing a theoretical basis for the development of efficient lignin-degrading bacterial resources.

## 2. Materials and Methods

### 2.1. Isolation of Lignin-Degrading Bacteria

Thirty termite samples were collected from the Agricultural Science Experimental Base of Hainan University (Haikou, China; 20.0615° N, 110.3313° E). The termite samples were washed 2–3 times with sterile physiological saline. The surface of the termite samples was then disinfected with 75% alcohol. The termite intestines were removed and placed in a sterilized crucible. Physiological saline (5 mL) was added, and the samples were ground thoroughly to prepare a termite intestinal homogenate. The homogenate was then diluted with sterile physiological saline 10^3^–10^6^ times, with three replicates for each dilution step. Two hundred microlitres of the diluted homogenate was then evenly spread on lignin culture medium, and the plates were incubated in a 37 °C constant-temperature incubator until distinct single colonies appeared. Individual colonies were selected according to morphology and size and subsequently cultured in liquid Luria–Bertani (LB) medium at 37 °C for 18 h. Then, the colonies were streaked onto a medium with lignin as the sole carbon source and cultured. This process was repeated three times to obtain strains that could grow stably in lignin medium. The obtained strains were rescreened with aniline blue medium. After fully culturing the strain in liquid LB medium, it was mixed with 30% glycerol in a 1:1 volume ratio, shaken evenly, and stored at −20 °C.

### 2.2. 16S rDNA Identification

A 1 mL sample of bacterial culture was added to a 1.5 mL centrifuge tube and centrifuged at 4 °C at 8000× *g* rpm for 1 min. The supernatant was removed, and the bacterial cells were collected and sent to Personalbio Biotechnology Co., Ltd. (Shanghai, China) for 16S rDNA identification. The 16S rDNA sequences obtained from the bacterial strain samples were uploaded to the NCBI database for BLAST(https://blast.ncbi.nlm.nih.gov, accessed on 20 March 2024) sequence alignment. The sequence with the highest homology to the bacterial strain was downloaded, and a similar genus and species were selected as references. The similarity was calculated using MEGA 7.0 software, and a phylogenetic tree was constructed.

### 2.3. Lignin Sample Preparation

Bacterial culture liquid (10^6^ CFU/mL) was inoculated into lignin liquid medium (pH 7.5), with each flask receiving 1 mL of inoculum. Uninoculated medium served as the control, with three replicates per group. The mediums were incubated in a constant temperature shaking incubator at 37 °C, 180 rpm for 7 days. Fifty millilitres of lignin solution was centrifuged at 25 °C at 3800× *g* rpm for 8 min. The supernatant was loaded into a dialysis bag for dialysis for 3 days to remove impurities, and then the lignin liquid was collected and freeze-dried to constant weight to obtain lignin solid samples. Each sample was mixed with 5 volumes of anhydrous ethanol, vortexed for 15 min, and then centrifuged at 8000× *g* rpm for 10 min to collect the precipitate. This washing procedure was repeated three times to remove soluble impurities from the medium and bacterial metabolites. Finally, the purified lignin samples were dried at 65 °C for 48 h and stored in a desiccator for subsequent experimental analyses.

### 2.4. Determination of Enzyme Activity and Lignin Degradation Rate

Bacterial laccase activity was assayed at 24 h intervals using the ABTS method [27], The assay was performed at 30 °C in a 3 mL reaction mixture containing 0.2 mL of 1 mmol/L ABTS, 2.7 mL of 0.1 mol/L sodium acetate buffer (pH 3.8), and 0.1 mL of crude enzyme extract. One unit of enzyme activity (U) was defined as the amount of enzyme required to oxidize 1 μmol of ABTS per minute. The bacterial lignin degradation rate was determined via the Prussian blue method [11], with uninoculated lignin solution serving as the control. The lignin degradation rate was quantified on 1, 3, 5, and 7 days. Each group underwent three parallel experiments.

### 2.5. Analysis of Lignin Structure

A small quantity of the lignin sample was uniformly coated on conductive adhesive, subjected to gold sputtering, and then observed under a field emission scanning electron microscope at an accelerating voltage of 10.0 kV.

The potassium bromide pressing method was used for sample preparation. Potassium bromide (100 mg) was mixed with 2 mg lignin sample, ground thoroughly under a drying lamp, and pressed into a plate. The infrared absorption curve was then observed in the 4000–400 cm^−1^ range.

Lignin sample (1.6 mg) was placed into a thermal gravimetric analyzer sample dish. The initial and final temperatures were set at 50 °C and 800 °C, respectively, with a heating rate of 20 °C/min and a nitrogen flow rate of 20 mL/min. The thermogravimetric and derivative thermogravimetric curves were subsequently analyzed.

For all the above experiments, uninoculated lignin samples served as the control group, with three replicates set for each group.

### 2.6. Whole-Genome Sequencing

A single colony of *B. cereus* BC-8 was transferred to LB broth and grown for 18 h. The cells were harvested by centrifugation at 4 °C and 8000× *g* rpm for 5 min. After discarding the supernatant, the pellet was resuspended in 1× PBS buffer and centrifuged again. This wash cycle was repeated two to three times until a clear supernatant was obtained. Finally, the washed cell pellet was flash-frozen in liquid nitrogen for 15 min and stored at −80 °C prior to shipment to Personalbio Biotechnology Co., Ltd. (Shanghai, China) for sequencing. Gene libraries with different insertion fragments were first constructed via the whole-genome shotgun approach. These libraries were then subjected to sequencing utilizing two complementary technologies: second-generation short-read sequencing on an Illumina NovaSeq platform and third-generation long-read single-molecule sequencing on a PacBio Sequel platform.

The whole genome sequence of *B. cereus* BC-8 was uploaded to the JSpeciesWS (https://jspecies.ribohost.com, accessed on 14 May 2024) website for Average nucleotide identity (ANI) analysis. An ANI value ≥ 95% was used as the cutoff for species demarcation. GeneMarkS 4.28 software was used to predict protein-coding genes in the whole genome sequence of *B. cereus* BC-8 [28]. Potential coding regions were predicted according to the nucleotide usage frequency across the genome. tRNAscan-SE was used to predict tRNA genes in the complete genome of *B. cereus* BC-8 [29], while Barrnap 3.1 software was employed to predict rRNA genes [30]. Prediction of other non-coding RNAs was primarily obtained via comparison with the Rfam database [31]. Carbohydrate-active enzymes in *B. cereus* BC-8 were annotated using the Carbohydrate Active Enzyme Database (CAZy), which primarily includes enzyme families associated with the degradation, modification, and synthesis of carbohydrates, including glycosidic bonds [32]. Annotation of carbohydrate-active enzymes in *B. cereus* BC-8 against the CAZy database employed a dual threshold: ORFs > 80 amino acids required an E-value ≤ 1 × 10^−5^, while those ≤80 amino acids required an E-value ≤ 1 × 10^−3^, with both requiring > 30% alignment coverage to the database sequence. Functional annotation of protein-coding genes in the complete genome sequence of *B. cereus* BC-8 was performed using the GO [33], eggNOG [34], and KEGG [35] databases. GO annotation for protein-coding genes was performed using the BLAST2GO 6.0 software with its default parameters. Protein sequences were aligned against the eggNOG database using DIAMOND BLASTP with an E-value cutoff of 1 × 10^−6^, and the eggNOG ortholog group identifier from the single best hit was assigned to each gene. KEGG Orthology (KO) and pathway annotations were obtained primarily using the KEGG Automatic Annotation Server (KAAS), the assigned KO terms were mapped to their corresponding KEGG pathways. Finally, CGView was used to generate a circular genome map [36].

## 3. Results

### 3.1. Identification of Strain BC-8

**Figure 1:**
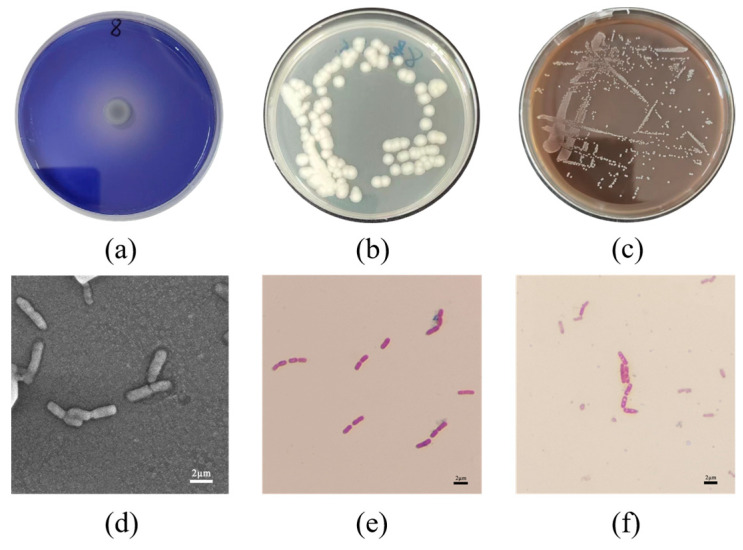
(**a**) Observation of hydrolyzed circles on aniline blue decolourisation medium. (**b**) Colony morphology of the *Bacillus cereus* BC-8 strain. (**c**) Colony morphology of the *B. cereus* BC-8 strain on lignin plates. (**d**) Microscopic morphology of *B. cereus* BC-8 strain. (**e**) Gram staining of *B. cereus* BC-8. (**f**) Observation of unstained spores.

**Figure 2:**
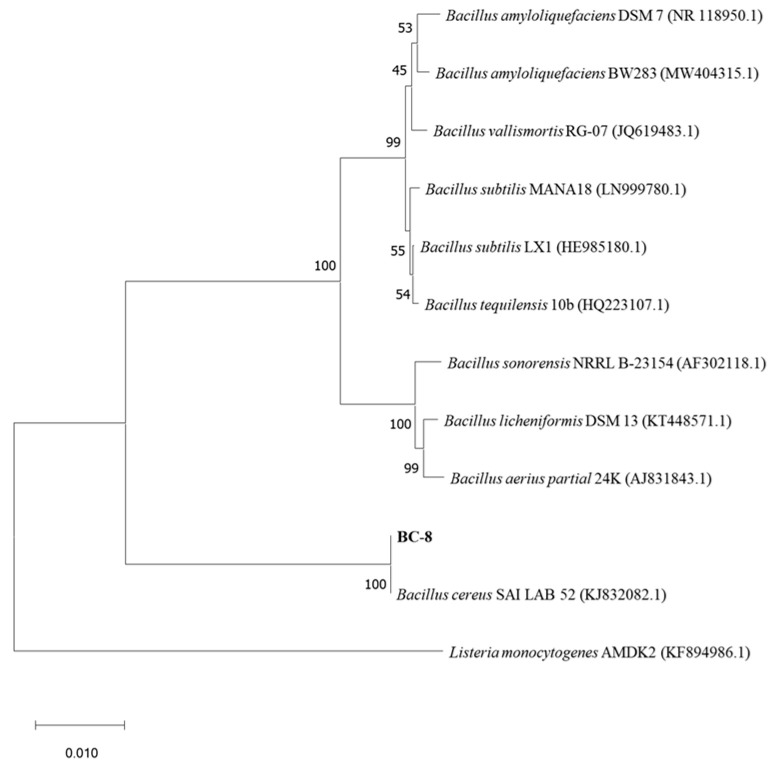
16S rDNA-sequence phylogenetic tree of *B. cereus* BC-8.

A total of twelve pure bacterial strains were isolated from the termite samples. Among these strains, strain BC-8 exhibited the highest lignin-degrading enzyme production ability, with the maximum ratio of D/d (D: 4.15 mm, d: 1.21 cm, D/d: 3.43; Figure 1a). Detailed D/d ratio data of all isolated strains can be found in Supplementary Material Table S1. Strain BC-8 exhibited white, rough colonies with waxy edges (Figure 1b) and grew stably on lignin plates (Figure 1c). SEM observations of the strain are shown in Figure 1d, with cell sizes of approximately 1.1 × 2.3 μm and a round rod-shaped morphology. Gram staining results indicated that the strain is Gram-positive (Figure 1e). Further observation revealed the presence of unstained spores (Figure 1f). The bacterial physiological and biochemical identification findings are presented in Supplementary Material Table S2. The phylogenetic tree of the strain is shown in Figure 2, revealing a similarity exceeding 99% with *Bacillus cereus.* Given the above results, the strain was named *B. cereus* BC-8.

### 3.2. Enzyme Activity and Lignin Degradation Rate of B. cereus BC-8

**Figure 3:**
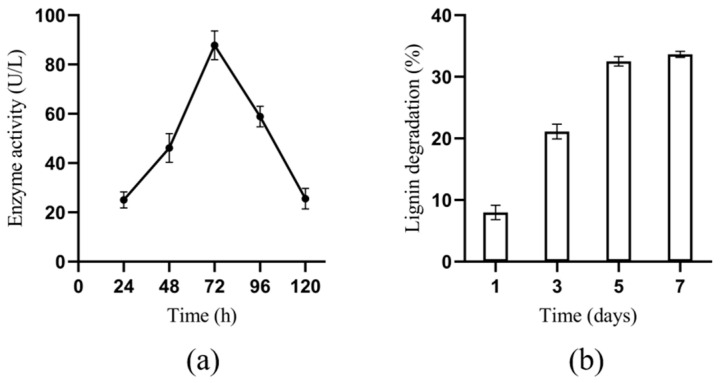
Laccase activity and lignin degradation rate of *B. cereus* BC-8. (**a**) Laccase activity. (**b**) Lignin degradation rate.

The laccase activity and lignin degradation rate of *B. cereus* BC-8 are shown in Figure 3. The laccase activity of *B. cereus* BC-8 first increased and then decreased, reaching a maximum of 87.8 U/L at 72 h, and then declined to 58.9 U/L at 96 h. The lignin degradation rate of *B. cereus* BC-8 increased continuously within 7 days, finally reaching 33.66%.

### 3.3. Scanning Electron Microscopy Observations

**Figure 4:**
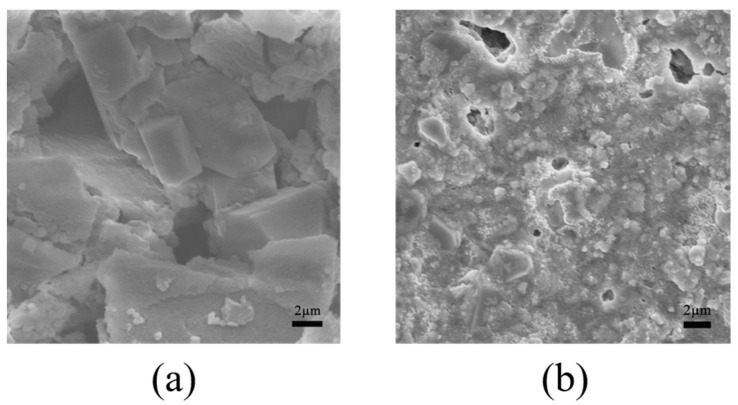
SEM image of lignin samples. (**a**) Control group. (**b**) The lignin samples were treated with *B. cereus* BC-8.

The SEM observations in lignin are shown in Figure 4. The lignin samples in the control group were distributed in blocks with neat edges and intact, smooth surface structures. Under the same magnification, the lignin samples treated with *B. cereus* BC-8 were mostly irregular in shape, with loose, porous surfaces and irregular edges.

### 3.4. Fourier Transform Infrared Spectroscopy Analysis

**Figure 5:**
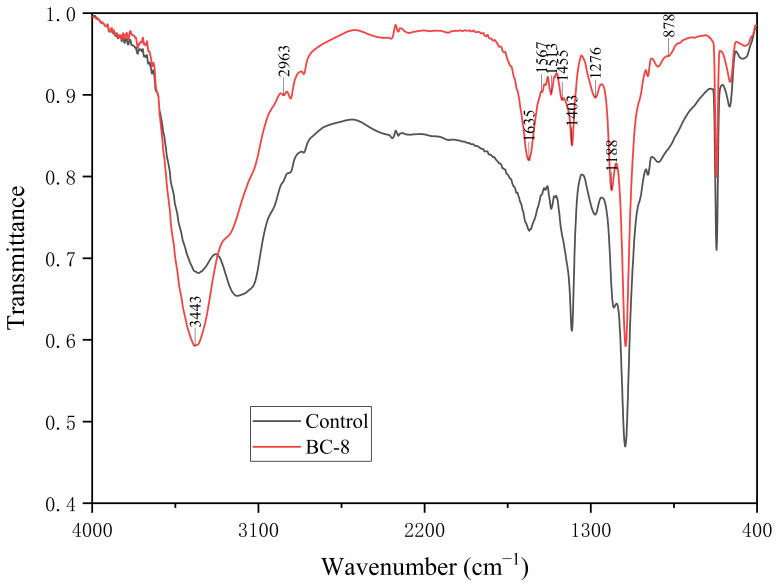
FTIR image of lignin samples.

The results of the FTIR spectroscopy analysis are shown in Figure 5. Compared with the control group, the treated group exhibited a shift in the absorption peak at 3443 cm^−1^ and new absorption peak at 1455 cm^−1^, the intensity of the absorption peaks at 1635 cm^−1^ and 1188 cm^−1^ increased, whereas the intensity of the absorption peak at 1403 cm^−1^ decreased. These changes are associated with the oxidation reaction of lignin side chains and the stretching vibrations of the aromatic ring skeleton in the lignin structure.

### 3.5. Thermal Gravimetric Analysis

**Figure 6:**
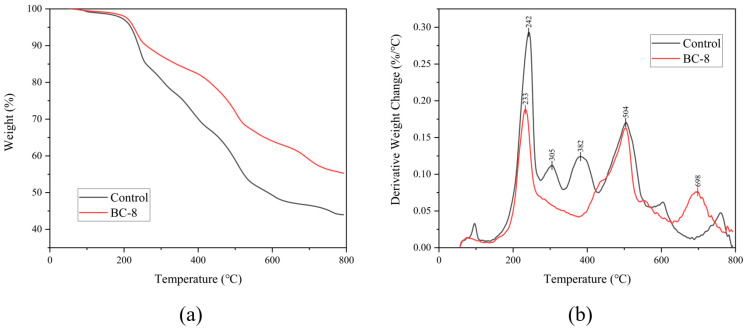
TGA plots of lignin samples. (**a**) Thermogravimetric curves. (**b**) Derivative thermogravimetric curves.

The results of the TGA are shown in Figure 6. The control lignin sample exhibited multiple weight loss peaks throughout the heating process, with the maximum occurring at 242 °C. Subsequently, two consecutive peaks appeared at 305 and 382 °C, followed by a significant one at 504 °C. Thereafter, the lignin mass loss tended to flatten, with the final remaining sample mass at 44.10%. Lignin samples treated with *B. cereus* BC-8 exhibited three distinct weight loss peaks at 233, 504, and 698 °C. Unlike in the control group, the mass loss rate of the *B. cereus* BC-8-treated samples remained relatively constant between 250 and 400 °C, with no weight loss peaks observed, and the final remaining sample accounted for 56.59%. Detailed TGA parameters are provided in Supplementary Material Table S3.

### 3.6. Whole-Genome Sequencing and Functional Annotation of B. cereus BC-8

**Figure 7:**
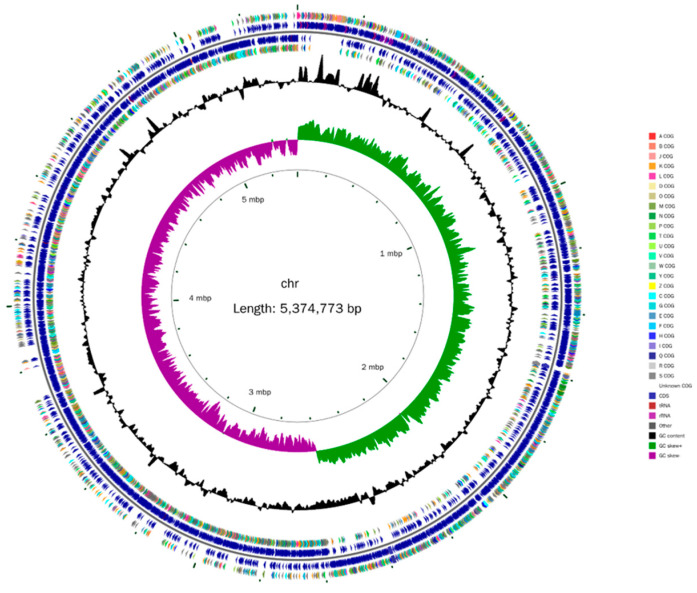
The circular genome map of *B. cereus* BC-8.

The whole genome map of *B. cereus* BC-8 is shown in Figure 7. The entire genome length is 5,374,773 bp, with a GC content of 35.34%. The genome contains 5440 open reading frames, 109 tRNAs, 52 rRNAs, and 127 ncRNAs. The specific results are shown in Supplementary Material Table S4.

The results of ANI analysis are shown in Table 1. The similarity between the whole genome sequence of *B. cereus* BC-8 and *Bacillus cereus* was more than 95%, and the similarity with *Bacillus cereus* INRA A3 was the highest, reaching 98.13%.

**Figure 8:**
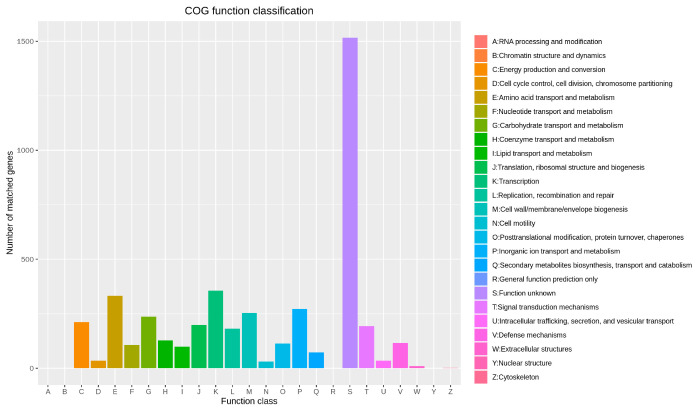
Diagram showing eggNOG functional classification of the *B. cereus* BC-8 genome.

Using the eggNOG database for annotation, 4488 genes were distributed across 21 categories of protein-coding genes. Genes with unknown functions were the most numerous, accounting for 27.89%, followed by genes related to transcription (355; 6.53%). Notably, the genome of *B. cereus* BC-8 contains 72 genes related to the biosynthesis, transport, and degradation of secondary metabolites, and 236 related to carbohydrate transport and metabolism (Figure 8).

Enzymes associated with lignin degradation were identified via eggNOG database annotation, as shown in Table 2. Among these, laccase, multicopper oxidase, and cytochrome P450 are involved in lignin degradation, with each enzyme encoded by one gene; catalase is an auxiliary enzyme in the lignin degradation process, with six encoding genes; monooxygenase, dioxygenase, oxidase, oxidoreductase, and dehydrogenase have 8, 30, 42, 29, and 110 encoding genes, respectively.

**Figure 9:**
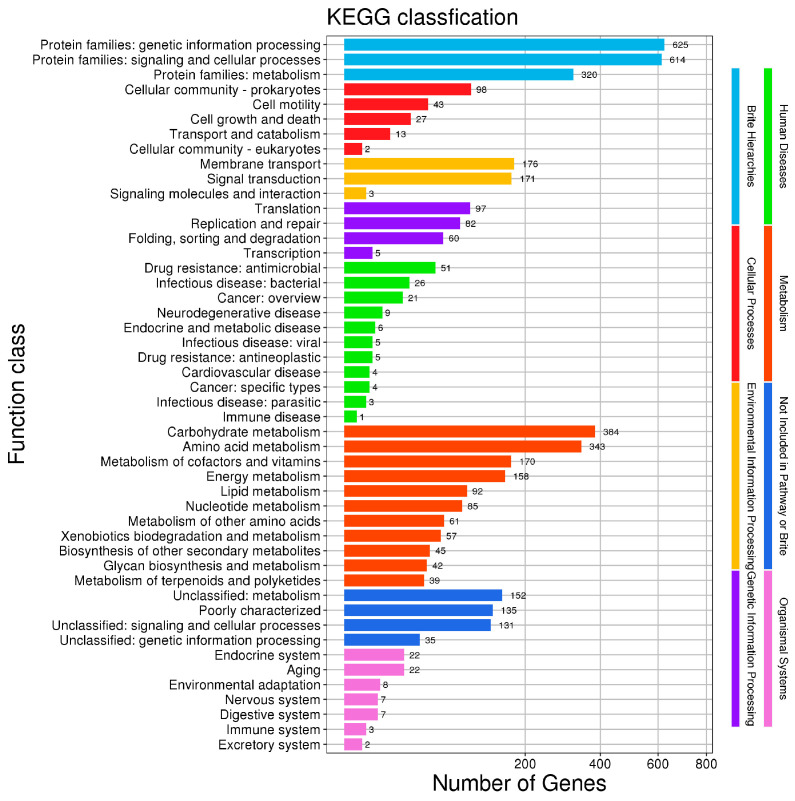
KEGG classification of the *B. cereus* BC-8 genome.

A total of 2373 genes were annotated in the KEGG database and classified into eight major categories and 47 subcategories. The largest numbers of genes were annotated in the protein family genetic information processing and signal transduction and cell process categories (625 and 614 genes, respectively). Genes related to carbohydrate metabolism (384 genes), amino acid metabolism (343 genes), signal transduction (171 genes), and membrane transport (176 genes) were also observed (Figure 9).

KEGG database annotation revealed pathways related to aromatic compound metabolism, as shown in Table 3. Among these, genes associated with phenylalanine metabolism and benzoic acid degradation are the most numerous, with 11 genes each, whereas 4 genes each were associated with chlorocyclohexane, chlorobenzene, and styrene degradation. Three genes were associated with xylene, naphthalene, and aminobenzoic acid degradation and cytochrome P450 metabolism. One gene was associated with nitrotoluene and ethylbenzene degradation.

### 3.7. Annotation Results of Carbohydrate-Active Enzymes

**Figure 10:**
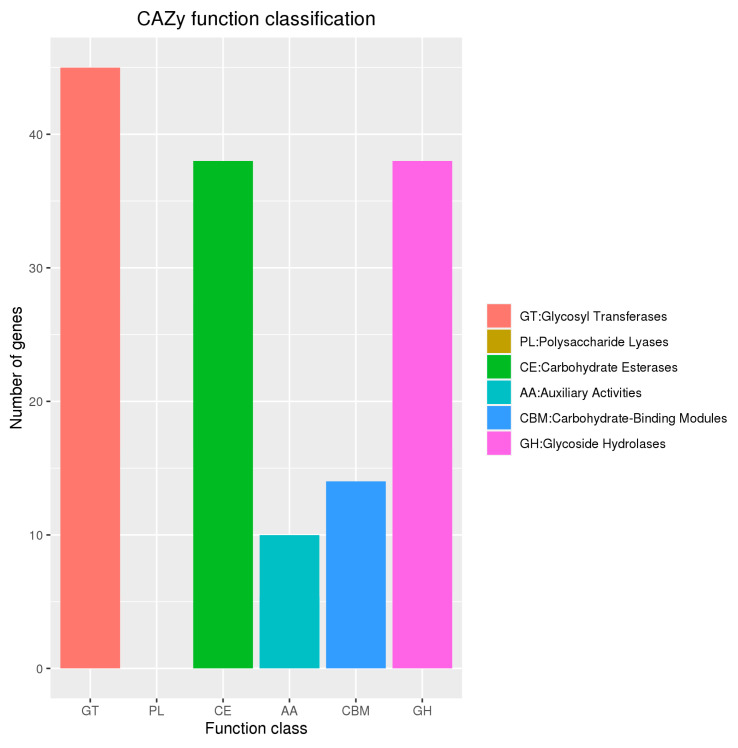
The number of genes belonging to carbohydrate-active enzyme families in *B. cereus* BC-8.

The annotation of the *B. cereus* BC-8 genome in the CAZy database is shown in Figure 10. Specifically, 45 genes were annotated as glycosyl transferases, 0 as polysaccharide lyases, 38 as carbohydrate esterases, 10 as auxiliary activities, 14 as carbohydrate-binding modules, and 38 as glycoside hydrolases.

In the annotation of carbohydrate-related enzymes, enzymes associated with lignin degradation are primarily classified under the auxiliary activity enzymes category. Therefore, genes related to lignin degradation should be identified in the annotation of auxiliary activity enzymes, as shown in Table 4. The genome of *B. cereus* BC-8 contains one laccase (EC 1.10.3.2) gene, two vanillyl alcohol oxidase (EC 1.1.3.38) genes, two 1,4-benzoquinone reductase (EC 1.6.5.6) or glucooligosaccharide oxidase (EC 1.1.3) genes, three chitooligosaccharide oxidase (EC 1.1.3) or cellooligosaccharide dehydrogcnase (EC 1.1.99) genes, and one monooxygenase (EC 1.14.99) gene.

## 4. Discussion

In this study, a lignin-degrading bacterium was isolated from termites. Morphological observation, physiological and biochemical tests, and Gram staining identified the strain as belonging to the *Bacillus* genus. *Bacillus* bacteria have been confirmed as important microorganisms in the lignin degradation process [37]. Previous studies have demonstrated that the ability of bacteria to produce extracellular enzymes can be detected using aniline blue medium [18]. By measuring the decolourisation effect of bacteria on aniline blue medium, strains with stronger enzyme-producing activity can be further screened. In this study, the ratio of the aniline blue decolourisation zone to the colony diameter was compared, successfully screening a bacterium with strong enzyme-producing activity. We constructed a phylogenetic tree based on 16S rDNA sequences to determine the specific species of the strain. Generally, strains with similarity greater than 97% are considered to belong to the same species [38]. According to the phylogenetic tree comparison results, the similarity of this bacterium to *B. cereus* exceeded 99%, thus confirming it as *B. cereus*. Mei et al. [39] reported that *Bacillus amyloliquefaciens* SL-7 exhibited a maximum laccase activity of 55.95 U/L and a lignin degradation rate of 28.55%, demonstrating a significant lignin-degrading capacity. Zhong et al. [40] isolated a *B*. *cereus* strain from buffalo rumen, which achieved a kraft lignin degradation rate of 25.9%. In comparison, *B. cereus* BC-8 exhibited a peak laccase activity of 87.8 U/L and a lignin degradation rate of 33.66%, demonstrating superior laccase activity and lignin degradation efficiency. *Rhodococcus* is considered a robust microorganism for lignin decomposition, with a lignin degradation rate of up to 19% [41]. Additionally, *Pseudomonas putida* is also an excellent lignin degrader, achieving a degradation rate of 30% [42]. This demonstrates that *B. cereus* BC-8 exhibits outstanding lignin degradation performance.

The lignin degradation capacity of the strain was investigated through lignin structural analysis. FE–SEM uses high-energy electrons to scan the sample surface, revealing its microscopic structure. Observing the surface structure of lignin before and after degradation using SEM is one of the key technical methods used to determine whether lignin can be degraded or modified by microorganisms [43]. In this study, lignin samples degraded by bacteria were observed using SEM and compared with undegraded lignin samples. Significant changes in the microscopic structure of lignin were observed. As a type of high-molecular-weight polymer, lignin has a highly compact surface structure, typically distributed in block-like or spherical shapes [16]. Due to its stable nature, lignin is difficult to degrade naturally. In the control group, after 7 days of blank treatment, the surface structure of the lignin samples remained intact and was only affected by physical and mechanical damage, with most of the lignin surface structure well-preserved in comparison with its original structure. In contrast, after treatment with *B. cereus* BC-8, the lignin surface structure exhibited pores, irregular edges, and numerous lignin particles with diameters less than 10 μm. This finding may be attributable to the secretion of lignin-degrading enzymes by the bacterium, which act on the lignin surface, leading to structural damage and a reduction in molecular weight. FTIR can visualize changes in chemical bonds before and after lignin degradation [44]. In this study, the infrared spectrum showed changes in the benzene ring side chains and aromatic skeleton structure of lignin after degradation. The peak at 3443 cm^−1^ corresponds to the characteristic position of the hydroxyl group on the benzene ring of lignin [45]. After treatment with *B. cereus* BC-8, the absorption peak at this position shifts, which is caused by the intramolecular stretching of the phenolic hydroxyl group, indicating that bacteria have damaged the hydroxyl groups of lignin. The phenolic hydroxyl groups affect the condensation degree of lignin, thereby reducing its overall stability. Additionally, changes in the 1600–600 cm^−1^ range are primarily associated with the stretching vibrations of the aromatic skeleton of lignin [46]. After bacterial degradation, the absorption peak at 1403 cm^−1^ in the lignin sample is weakened, related to the oxidation reactions of the lignin side chains and the stretching vibrations of the aromatic ring skeleton, a new absorption peak appeared at 1455 cm^−1^, indicating that the lignin structure changed, and the absorption peaks at 1635 cm^−1^ and 1188 cm^−1^ are enhanced, which are related to the C=O stretching vibration and the stretching vibration of the aromatic ring skeleton, respectively [47,48]. TGA can detect changes in material mass with temperature. The more stable the sample is, the higher the temperature required for decomposition will be, making TGA crucial for studying lignin thermal stability. This approach can reveal changes in chemical structure after lignin degradation [49]. Ether bonds are the primary connecting bonds between lignin structural units [50]. The TGA results revealed that the control group lignin samples exhibited two weight loss peaks at 250–400 °C. This temperature range primarily involves the breaking of β-O-4 ether bonds in the lignin structure [51]. Therefore, this finding indicates that the control group lignin samples contained a high content of β-O-4 ether bonds, resulting in poor thermal stability at 250–400 °C. After treatment with *B. cereus* BC-8, the derivative thermogravimetry curve of lignin became relatively flat at 250–400 °C, indicating that the structure of lignin had changed within this temperature range, resulting in improved thermal stability. The lower content of β-O-4 chemical bonds in the lignin samples may be due to the bacterial degradation of lignin, which disrupted the β-O-4 ether bonds. The degradation of the aromatic skeleton of lignin occurs around 400 °C [52]. Unlike in the TGA results of the control group, it was found that *B. cereus* BC-8 caused some damage to the aromatic skeleton of lignin, resulting in higher thermal stability of the lignin samples at around 400 °C than that observed in the control group. Additionally, the demethylation reaction of lignin, i.e., the release of methoxy groups from the aromatic rings of lignin, occurs within the temperature range of 400–550 °C [53]. A comparison revealed that lignin samples exhibited weight loss peaks within the 400–550 °C temperature range before and after degradation, indicating that *B. cereus* BC-8 exerted little effect on the demethylation reaction of lignin.

Whole-genome sequencing was performed to investigate the biological characteristics of *B. cereus* BC-8 at the molecular level and its regulatory mechanisms in lignin degradation. Following sequencing, the total gene sequence length of the *B. cereus* BC-8 strain was found to be 5,374,773 bp, containing 5440 open reading frames, with a GC content of 35.34%. This result is similar to the genome of *B. cereus* screened from buffalo rumen by Zhong et al. [40]. The Cazy database annotated the hydrolytic enzyme activity genes of *B. cereus* BC-8, indicating that it possesses multiple auxiliary enzyme genes. Further identification of enzymes related to lignin degradation revealed that *B. cereus* BC-8 encodes laccase, vanillyl alcohol oxidase, 1,4-benzoquinone reductase, glucooligosaccharide oxidase, chitooligosaccharide oxidase, cellooligosaccharide dehydrogcnase, and monooxygenase genes. Among these active enzymes, laccase is a multicopper oxidase with broad substrate specificity, capable of catalyzing the oxidation of phenolic hydroxyl groups in lignin. It serves as a key oxidative enzyme in the bacterial degradation of lignin. The shift at 3443 cm^−1^ in the infrared spectrum also indicates this point [6]. Vanillyl-alcohol oxidase belongs to the AA4 family (4-phenol oxidase family) [54] and can catalyze the conversion of lignin to vanillin through the oxidation of side chains, which is associated with the weakening of the absorption peak at 1403 cm^−1^ in the infrared spectrum [55]. Whereas 1,4-benzoquinone reductase, glucooligosaccharide oxidase, chitooligosaccharide oxidase, cellooligosaccharide dehydrogcnase, and monooxygenase play auxiliary roles in lignin degradation [56]. According to the KEGG database annotation results, three *B. cereus* BC-8 genes are associated with the aminobenzoic acid degradation pathway. Studies have shown that the aminobenzoic acid degradation pathway includes two branches of lignin metabolism: the methyl gallic acid degradation pathway and the protocatechuic acid degradation pathway [57]. Vanillin is an important intermediate in both of these lignin biodegradation pathways, and the vanillin alcohol oxidase gene present in *B. cereus* BC-8 may play a key role in both lignin degradation pathways. Masai et al. [58] reported that in the lignin protocatechuic acid degradation pathway, vanillin oxidase first catalyzes protocatechuic acid and ultimately produces oxalacetic acid and pyruvic acid. Thus, it can be inferred that *B. cereus* BC-8 participates in both lignin degradation pathways. Additionally, three *B. cereus* BC-8 genes were annotated as part of the cytochrome P450 metabolic pathway, which is associated with lignin degradation. In the eggNOG database, one laccase-encoding gene, one polyphenol oxidase-encoding gene, and one cytochrome P450 gene were identified. The cytochrome P450 can catalyze the hydroxylation and ring-opening reactions of aromatic rings, which corresponds to the stretching vibrations of the aromatic skeleton in the 1600–600 cm^−1^ range of the infrared spectrum [59]. Additionally, monooxygenases and dioxygenases have been shown to participate in lignin degradation processes [56].

Regarding biosafety, *B. cereus* is a well-known foodborne pathogen. It is generally recognized that a concentration exceeding 10^4^ CFU/g in food can cause food poisoning in both animals and humans, leading to symptoms such as diarrhea and vomiting. International food safety guidelines often set the satisfactory threshold for *B. cereus* in ready-to-eat foods at <10^3^ CFU/g [60]. However, it is important to note that the *B. cereus* group exhibits significant strain diversity. Certain strains possess probiotic properties and are utilized as oral supplements for humans and livestock. These probiotic strains function by promoting the growth of beneficial gut microbiota, alleviating intestinal inflammation, and inhibiting the colonization of pathogens [61]. *B. cereus* BC-8 holds great potential for industrial applications, particularly in recycling agricultural waste such as straw and converting lignin into biofuels. However, the potential pathogenicity of some strains raises concerns about environmental contamination risks in large-scale, open industrial processes. Therefore, to safely harness the capabilities of *B. cereus* BC-8 for industrial applications, further research into its pathogenicity and probiotic effects is necessary. Concurrently, the key lignin-degrading enzyme genes, such as the laccase gene, can be cloned and heterologously expressed in Generally Recognized As Safe (GRAS) microbial hosts like *Pichia pastoris* [62]. This approach decouples the valuable enzymatic function from the biosafety concerns associated with the native bacterial host, providing a safer alternative for industrial implementation.

## 5. Conclusions

In this study, a strain of *B. cereus* with high lignin degradation ability was isolated from termites, designated *B. cereus* BC-8. This strain can degrade lignin by disrupting its aromatic skeleton, side chains, and β-O-4 ether bonds, thereby reducing the structural stability of lignin. *B. cereus* BC-8 has the coding genes of laccase, cytochrome P450, vanillin oxidase and other lignin-degrading enzymes, as well as key genes involved in lignin-related degradation pathways such as aminobenzoic acid metabolism. Among these, laccase serves as a key oxidase in the lignin degradation process of *B. cereus* BC-8, and its encoding gene represents a promising target for future genetic engineering applications. Furthermore, *B. cereus* BC-8 offers significant potential for applications in the recycling of straw waste and the conversion of lignin into biofuels.

## Figures and Tables

**Table 1:** Average nucleotide identity analysis of *B. cereus* BC-8.

Genome	ANIm (%)
*Bacillus cereus* INRA A3	98.13
*Bacillus cereus* FORC_005	98.10
*Bacillus cereus* MIT0214	98.09
*Bacillus cereus* F3175-03	97.17
*Bacillus cereus* tsu1	97.13
*Bacillus cereus* ATCC 10876	97.01
*Bacillus cereus* CMCC P0011	96.87
*Bacillus cereus* B4158	96.74
*Bacillus cereus* B4080	96.61

**Table 2:** Enzymes related to lignin degrading in functional annotation of eggNOG.

Enzymes	Number of Genes	Gene IDs
Laccase	1	chr_1806
Multicopper oxidase	1	chr_3871
Cytochrome P450	1	chr_2849
Monooxygenase	8	chr_1023, chr_1645, chr_2568
Dioxygenase	30	chr_231, chr_233, chr564
Catalase	6	chr_749, chr_792, chr_1127; chr_2888, chr_4748, chr_4749
Oxidase	42	chr_173, chr_183, chr_651
Oxidoreductase	29	chr_267, chr_370, chr_500
Dehydrogenase	110	chr_171, chr_312, chr_325

Note: Only three IDs are listed in the table when more than three genes are found.

**Table 3:** The number of genes of *B. cereus* BC-8 enriched in KEGG pathways related to aromatic compound metabolism.

Pathways	Encoding	Number of Genes
Phenylalanine metabolism	00360	11
Chlorocyclohexane and chlorobenzene degradation	00361	4
Benzoate degradation	00362	11
Xylene degradation	00622	3
Naphthalene degradation	00626	3
Aminobenzoate degradation	00627	3
Nitrotoluene degradation	00633	1
Ethylbenzene degradation	00642	1
Styrene degradation	00643	4
Metabolism of xenobiotics by Cytochrome P450	00980	3

**Table 4:** Annotated genes encoding lignin-degrading enzymes in *B. cereus* BC-8.

Auxiliary Activity	Gene Symbols	Enzymes	Evalue
AA1_3	*mmcO*	laccase (EC 1.10.3.2)	1.3 × 10^−39^
AA4	*glcD*; *ldhd*	vanillyl alcohol oxidase (EC 1.1.3.38)	4.7 × 10^−31^; 7.4 × 10^−24^
AA6	*ykuN*; *ywqN*	1,4-benzoquinone reductase (EC 1.6.5.6); glucooligosaccharide oxidase (EC 1.1.3)	2.7 × 10^−6^; 2.7 × 10^−7^
AA7	*yitY*; *ygaK*; *Rv1771*	chitooligosaccharide oxidase (EC 1.1.3); cellooligosaccharide dehydrogcnase (EC 1.1.99)	1.9 × 10^−12^; 1.4 × 10^−100^; 8.6 × 10^−20^
AA10	*gbpA*	monooxygenase (EC 1.14.99)	6.2 × 10^−56^

## Data Availability

The original contributions presented in this study are included in the article/Supplementary Material. Further inquiries can be directed to the corresponding author.

## Supplementary Materials

The following supporting information can be downloaded at: https://www.mdpi.com/article/10.3390/microorganisms14010054/s1, Table S1: Results of aniline blue decolorization ring. Table S2: Physiological and biochemical characteristics of *B. cereus* BC-8. Table S3: Parameters from the TGA plot. Table S4: Results of non coding RNA in *B. cereus* BC-8 genome.
