# Supplementary material for: Whole-Genome Analysis and Lignin Degradation Characterization of Termite-Derived Bacillus cereus BC-8
Source: Microorganisms. 2025 Dec 26;14(1):54. doi: 10.3390/microorganisms14010054 (PMC12843926; doi:10.3390/microorganisms14010054)
Supplement: Supplementary file 1 [file microorganisms-14-00054-s001.zip › microorganisms-4034166-supplementary.pdf]

**Table S1.** Results of aniline blue decolorization ring.

| Number | D ( cm ) | d ( cm ) | D/d |
|---|---|---|---|
| 1 | 3.11 | 1.02 | 3.05 |
| 2 | 3.71 | 1.15 | 3.23 |
| 3 | 3.31 | 1.13 | 2.93 |
| 4 | 2.83 | 1.01 | 2.80 |
| 5 | 3.38 | 1.10 | 3.07 |
| 6 | 4.01 | 1.27 | 3.16 |
| 7 | 3.23 | 1.05 | 3.08 |
| 8 | 4.15 | 1.21 | 3.43 |
| 9 | 4.12 | 1.38 | 2.99 |
| 10 | 3.40 | 1.41 | 2.41 |
| 11 | 4.01 | 1.33 | 3.02 |
| 12 | 3.83 | 1.25 | 3.06 |

**Table S2.** Physiological and biochemical characteristics of *B. cereus* BC-8.

| Test | Results |
|---|---|
| Catalase test | + |
| Nitrate reduction test | + |
| Hydrogen sulphide qualitative test | − |
| Starch hydrolysis test | + |
| Mannitol fermentation | − |
| pH=3 | − |
| pH=7 | + |
| pH=11 | + |

Note: + means positive reaction, − means negative reaction.

**Table S3.** Parameters from the TGA plot.

| Parameter | Temperature ( ℃ ) | Weight ( % ) | |
|---|---|---|---|
| | | Control | BC-8 |
| Onset | 50 | 100 | 100 |
| DTG peak | 233 | 91.87±2.10 | 94.67±1.27 |
| | 242 | 88.46±1.92$^{a}$ | 92.63±0.74$^{b}$ |
| | 305 | 80.71±0.53$^{a}$ | 88.17±1.00$^{b}$ |
| | 382 | 72.50±1.72$^{a}$ | 84.33±1.30$^{b}$ |
| | 504 | 58.27±2.04$^{a}$ | 71.22±0.90$^{b}$ |
| | 698 | 46.54±0.35$^{a}$ | 59.69±0.66$^{b}$ |
| Residual mass | 792 | 44.10±0.36$^{a}$ | 56.59±0.53$^{b}$ |

Note: Data are means of three samples; means with different letters in the same row (a and b) differ significantly ($p < 0.05$).

**Table S4.** Results of non coding RNA in *B. cereus* BC-8 genome.

| Type | Number | Average length ( bp ) | Total length ( bp ) | Percentage of genome ( % ) |
|---|---|---|---|---|
| 5S rRNA | 14 | 109 | 1526 | 0.0284 |
| 16S rRNA | 14 | 1550 | 21700 | 0.4037 |
| 23S rRNA | 14 | 2919 | 40866 | 0.7603 |
| tRNA | 109 | 77 | 8428 | 0.1568 |
| ncRNA | 127 | 169 | 21569 | 0.4013 |
